# Bromodomain and Extra-Terminal Inhibitor BMS-986158 Reverses Latent HIV-1 Infection In Vitro and Ex Vivo by Increasing CDK9 Phosphorylation and Recruitment

**DOI:** 10.3390/ph15030338

**Published:** 2022-03-10

**Authors:** Xu-Sheng Huang, Ren-Rong Tian, Meng-Di Ma, Rong-Hua Luo, Liu-Meng Yang, Guang-Hui Peng, Mi Zhang, Xing-Qi Dong, Yong-Tang Zheng

**Affiliations:** 1 Key Laboratory of Bioactive Peptides of Yunnan Province/Key Laboratory of Animal Models and Human Disease Mechanisms of the Chinese Academy of Sciences, KIZ-CUHK Joint Laboratory of Bioresources and Molecular Research in Common Diseases, Center for Biosafety Mega-Science, Kunming Institute of Zoology, Chinese Academy of Sciences, Kunming 650223, China; huangxs317@163.com (X.-S.H.); tianrenrong@mail.kiz.ac.cn (R.-R.T.); mamengdi@mail.kiz.ac.cn (M.-D.M.); luorh@mail.kiz.ac.cn (R.-H.L.); lmyang@mail.kiz.ac.cn (L.-M.Y.); 2 University of Chinese Academy of Sciences, Beijing 100049, China; 3 Yunnan Provincial Infectious Diseases Hospital/Yunnan Provincial Care Center for AIDS, Kunming 650301, China; pengguanghui2021@126.com (G.-H.P.); zm050306@sohu.com (M.Z.); dongxq99@vip.sina.com (X.-Q.D.)

**Keywords:** HIV-1, BET, BMS-986158, latency reversing agent, latent reservoir

## Abstract

Latent reservoir persistence remains a major obstacle for curing human immunodeficiency virus type 1 (HIV-1) infection. Thus, strategies for the elimination of latent HIV-1 are urgently needed. As a bromodomain and extra-terminal (BET) inhibitor, BMS-986158 has been used in clinical trials for advanced solid tumors and hematological malignancies. Here, we found that BMS-986158 reactivated latent HIV-1 in three types of HIV-1 latency cells in vitro, and in combination antiretroviral therapy (cART)-treated patient-derived peripheral blood mononuclear cells ex vivo, without influencing global immune cell activation. BMS-986158 reactivated latent HIV-1 by increasing phosphorylation of CDK9 at Thr186 and promoting recruitment of CDK9 and RNA polymerase II to the HIV-1 long terminal repeat in J-Lat cells. Furthermore, BMS-986158 exerted strong synergism in reactivating latent HIV-1 when combined with prostratin and vorinostat and enhanced the antiviral activity of anti-HIV-1 drugs. Finally, BMS-986158 showed antiviral activity in an HIV-1 acute infection model, possibly by arresting the cell cycle in infected cells. Thus, these results suggest that BMS-986158 is a potential candidate for AIDS/HIV-1 therapy.

## 1. Introduction

Acquired immunodeficiency syndrome (AIDS) is an incurable disease caused by human immunodeficiency virus (HIV) infection. Although combination antiretroviral therapy (cART) can suppress HIV replication to undetectable levels and promote immune system reconstruction, the virus can persist in various cells and tissues of HIV-infected patients and may not be eliminated [1,2]. The integrated and transcriptionally silent proviruses harbored in resting CD4^+^ T cells are considered as primary latent HIV reservoirs [3]. Latent reservoirs established in early infection, which can persist lifelong under ongoing cART, remain a huge obstacle for treatment of HIV infection [4].

The “shock and kill” strategy, which aims to reactivate and then purge the latent reservoir with cART, has gained attention in recent years [5,6]. The first challenge of this strategy is to identify potent latency reversing agents (LRAs) to reactivate the latent virus. Many such agents have been investigated in vitro and ex vivo, with several reaching the clinical trial stage [7,8,9,10,11]. LRAs can be divided into three groups: (1) transcription initiation promoting agents, including histone deacetylase (HDAC), histone methyltransferase (HMT), and DNA methyltransferase (DNMT) inhibitors and protein kinase C (PKC) activators; (2) transcription elongation promoting agents, including positive transcription elongation factor b (P-TEFb) activators; and (3) post-transcription modifying agents [12].

As a well-conserved class of transcriptional regulators differentiated by the presence of tandem bromodomains, conserved domains, and extra-terminal domains, the human bromodomain and extra-terminal (BET) family of proteins has received increasing attention [13,14,15]. The well-studied bromodomain-containing BET protein BRD4 is considered a major impediment to latency reactivation due to its inhibition of Tat-transactivation [16,17,18]. The BET protein BRD2 is also implicated in HIV-1 transcription in latent cells [19]. Several recent studies have reported that BET inhibitors, including JQ1, OTX015, and apabetalone, can reactivate HIV-1 in different cell models and patient-derived resting CD4^+^ T cells [20,21,22]. BMS-986158, another BET inhibitor, has been applied in several clinical trials for advanced solid tumors and hematological malignancies [23,24].

In the current study, we evaluated the effects of BMS-986158 on HIV-1 latency. Results indicated that BMS-986158 efficiently reactivated latent HIV-1 by inducing cyclin-dependent kinase 9 (CDK9) phosphorylation and promoting CDK9 and RNA polymerase II (RNAP II) recruitment to stimulate HIV-1 transcription elongation. The reactivation activity of BMS-986158 was much higher than that of JQ1. Furthermore, the reactivation effect was potently enhanced by combining BMS-986158 with prostratin or vorinostat (VOR; also known as suberanilohydroxamic acid (SAHA)). In consideration of future clinical trials, we demonstrated that anti-HIV-1 drugs did not influence the reactivation activity of BMS-986158, while BMS-986158 enhanced the antiviral activity of these drugs. Our results also indicated that BMS-986158 induced latent HIV-1 expression in peripheral blood mononuclear cells (PBMCs) from individuals with suppressive ART, while displaying minimal toxicity and detrimental effects on T cell activation.

## 2. Results

### 2.1. BMS-986158 Reverses HIV-1 Latency In Vitro and Ex Vivo

**Figure 1:**
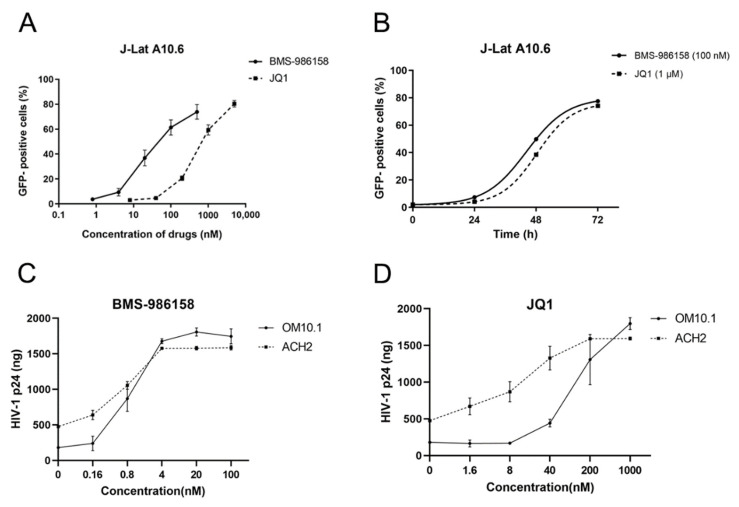
Reactivation activity of BMS-986158. (**A**) J-Lat cells were treated with gradient-diluted BMS-986158 and JQ1 for 48 h and percentage of GFP-positive cells was measured by flow cytometry. (**B**) J-Lat cells were treated with BMS-986158 and JQ1 for different times (0, 24, 48, and 72 h) and percentage of GFP-positive cells was measured. (**C**,**D**) OM10.1 and ACH2 cells were treated with double-diluted BMS-986158 or JQ1 for 48 h and quantity of HIV-1 p24 in cell culture supernatant was measured by ELISA. All data represent the mean ± SD of three independent experiments.

To determine the potential of BMS-986158 to reverse HIV-1 latency, an established HIV-1 latent J-Lat A10.6 cell line, which bears integrated HIV-1 long terminal repeat (LTR)-Tat and green fluorescent protein (GFP) genes, was treated with different concentrations of BMS-986158 (0.8–500 nM) for 48 h or with 100 nM BMS-986158 for different times. The percentage of reactivated cells (GFP-positive cells) increased in a dose-dependent and time-dependent manner after BMS-986158 and JQ1 treatment (Figure 1A,B). The reactivation activity of BMS-986158 was better than that of JQ1, even though the concentration of BMS-986158 was one-tenth that of JQ1. Similarly, the 50% of maximal effect (EC_50_) value of BMS-986158 was 30.82 nM, which was 17.28 times lower than that of JQ1 (600.03 nM).

To confirm the reactivation ability of BMS-986158, other established HIV-1 latent cell lines, i.e., ACH2 and OM10.1, which contained competent viral replication capacity, were treated with serially diluted BMS-986158 or JQ1 for 48 h, after which the relative expression of HIV-1 p24 in the cell culture supernatant was detected. Results showed that BMS-986158 reversed HIV-1 latency dose dependently. After treatment with 100 nM BMS-986158, HIV-1 p24 expression increased by 5–10 times, higher than the expression after treatment with 1 μM JQ1 (Figure 1C,D).

To confirm the reactivation ability of BMS-986158, we tested the effects of BMS-986158 on cell associated (CA) HIV-1 RNA in PBMCs isolated from HIV-1-infected individuals receiving long-term ART. After treatment with BMS-986158 or JQ1 for 24 h, the expression of CA HIV-1 RNA increased in 12 (BMS986158) and 11 (JQ1) of the 20 individuals, respectively (Table 1), indicating that BMS-986158 reactivated latent HIV-1 during ART ex vivo.

### 2.2. BMS-986158 Displays Low Toxicity in PBMCs and J-Lat Cells

**Figure 2:**
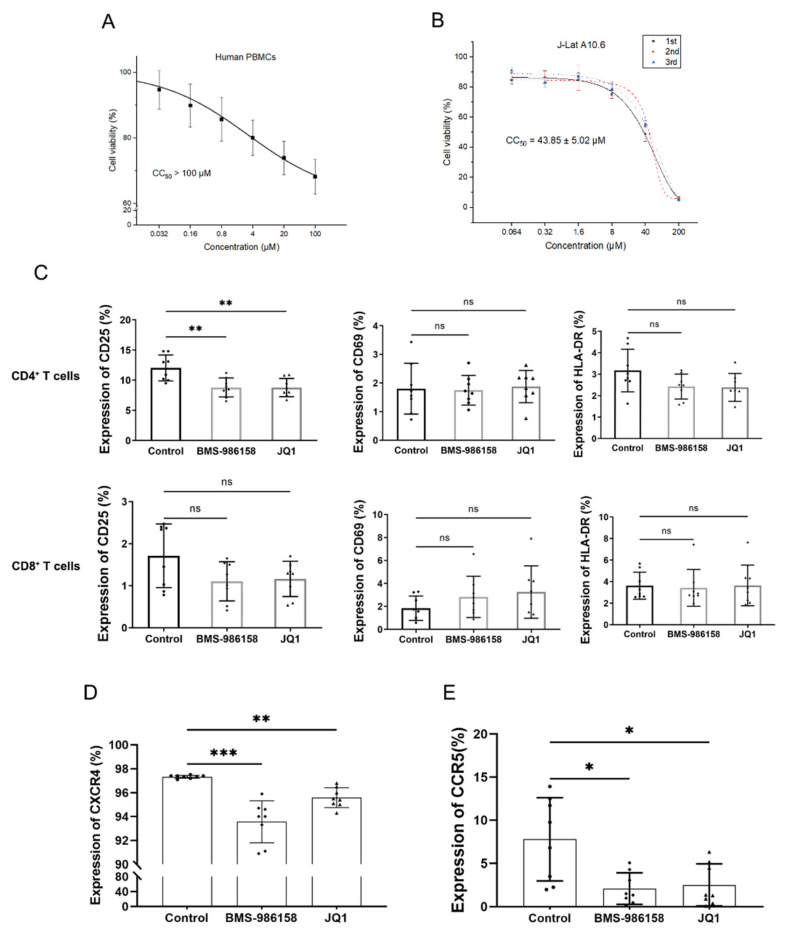
Toxicity of BMS-986158. (**A**,**B**) Cytotoxicity of BMS-986158 in PBMCs and J-Lat cells. PBMCs and J-Lat cells were treated with gradient-diluted BMS-986158 for 48 h and cell viability was measured by MTT. Data represent the mean ± SD of three independent experiments. (**C**) Effect of BMS-986158 on T cell activation. PBMCs from eight donors were treated with BMS-986158 or JQ1 for 48 h and stained with anti-CD4, anti-CD8, anti-CD25, anti-CD69, and anti-HLA-DR antibodies to measure expression by flow cytometry. (**D**,**E**) Effect of BMS-986158 on expression of HIV-1 co-receptors CXCR4 and CCR5. CD4^+^ T cells in PBMCs from eight donors were treated with BMS-986158 or JQ1 for 48 h and stained with anti-CXCR4 or anti-CCR5 antibodies to measure expression by flow cytometry. Single independent experiment was including for each donor (*n* = 8), data represent the mean ± SD. The statistically significant difference between control and BMS-986158, and control and JQ1 treatment group were analyzed using one-way ANOVA followed by Dunnett’s multiple comparisons test (**C**) and Dunnett’s T3 multiple comparisons test (**D**,**E**). (* *p* < 0.05; ** *p* < 0.01; *** *p* < 0.001; ns, no significance).

In consideration of clinical application, effective LRAs should be highly potent and minimally cytotoxic. To verify the cytotoxicity of BMS-986158, we treated PBMCs and J-Lat cells with gradient-diluted BMS-986158 for 48 h. As shown by MTT assay, PBMC viability decreased by 10% after treatment with BMS-986158 at an active concentration, with a 50% cytotoxicity concentration (CC_50_) > 100 μM (Figure 2A). The cytotoxicity of BMS-986158 in the J-Lat cells was larger than that in the PBMCs, with a CC_50_ of 43.85 μM (Figure 2B). Thus, BMS-986158 showed little toxicity in J-Lat cells and PBMCs at an active concentration.

Furthermore, regarding clinical treatment, LRAs should not induce non-specific immune activation. To explore whether BMS-986158 can induce cellular activation, PBMCs were treated with BMS-986158 for 48 h and then collected to determine the expression of cell surface activation markers (including CD25, CD69, and HLA-DR) by flow cytometry. Results showed that BMS-986158 did not up-regulate activation markers in CD4^+^ T and CD8^+^ T cells (Figure 2C).

Some LRAs, such as prostratin, can down-regulate cell surface receptors that mediate HIV entry [25]. Our results showed that BMS-986158 moderately down-regulated the expression of the CXCR4 and CCR5 co-receptors in the CD4^+^ T cells (Figure 2D,E), indicating that BMS-986158 does not increase potential de novo HIV-1 infection.

### 2.3. BMS-986158 Shows a Strong Inhibitory Effect on BRD4 Protein

**Figure 3:**
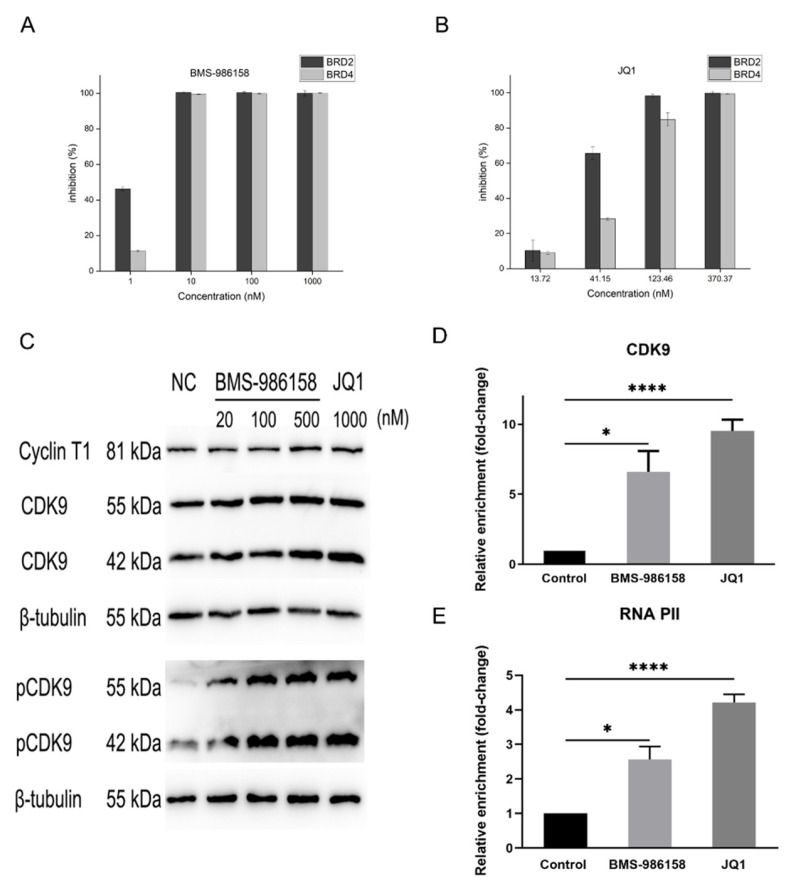
BMS-986158 reactivates latent HIV-1 by activating P-TEFb and promoting recruitment of P-TEFb and RNAP II. (**A**,**B**) BMS-986158 inhibited BRD2/4 activity in cell-free reaction system. Different concentrations of BMS-986158 or JQ1 were added to the system containing BRD2/4 and substrate, and inhibition was measured by homogeneous time-resolved fluorescence (HTRF) binding assay. (**C**) BMS-986158 significantly increased phosphorylation of CDK9. J-Lat cells were treated with different concentrations of BMS-986158 or JQ1 for 48 h and cell lysates were collected to measure expression of beta-tubulin, cyclin T1, CDK9, and phosphor-CDK9 by western blotting. (**D**,**E**) BMS-986158 promoted recruitment of CDK9 and RNAP II to HIV-1 LTR. J-Lat cells were treated with BMS-986158 or JQ1 for 6 h and cross-linked DNA-protein complexes were extracted to measure relative enrichment of CDK9 and RNAP II Ser2P on HIV-1 LTR by ChIP. All data represent the mean ± SD of three independent experiments. The statistically significant difference between control and BMS-986158, and control and JQ1 treatment group were analyzed using Kruskal–Wallis test followed by Dunn’s multiple comparisons test (* *p* < 0.05; **** *p* < 0.0001).

Although BMS-986158 is a BET protein target-based compound, whether it can inhibit BRD2/4 activity directly has not yet been reported. Therefore, we evaluated the inhibitory effects of BMS-986158 on BRD2/4 activity using the homogeneous time-resolved fluorescence (HTRF) binding assay. BMS-986158 almost completely inhibited BRD2 and BRD4 activity at 10 nM, with 50% inhibitory effect (IC_50_) values of 1.17 nM and 2.75 nM, respectively (Figure 3A,B). JQ1 was used as a control, with IC_50_ values of 30.18 nM (BRD2) and 62.71 nM (BRD4). Thus, these results imply that BMS-986158 is a better BRD2/4 inhibitor than JQ1.

### 2.4. BMS-986158 Promotes HIV Transcription via Activation of P-TEFb

P-TEFb is a transcription elongation factor consisting of two subunits, i.e., cyclin T1 and CDK9, which play important roles in the transcription of HIV [26]. The phosphor-CDK9 subunit of P-TEFb promotes HIV-1 transcription elongation via phosphorylation of the carboxyterminal domain (CTD) of RNAP II after recruitment to the HIV-1 LTR by Tat [27]. To verify the effects of BMS-986158 on P-TEFb activation, we treated J-Lat cells with different concentrations of BMS-986158 and collected the cell lysates for western blot assay. Results showed that BMS-986158 treatment did not significantly change CDK9 expression, but significantly increased phosphor-CDK9 expression, similar to JQ1 (Figure 3C). These results indicate that BMS-986168 can promote CDK9 activation.

BRD4 is a repressor of HIV transcription and competes with HIV Tat for P-TEFb recruitment [28]. To verify the effects of BMS-986158 on P-TEFb recruitment, J-Lat cells were treated with BMS-986158 or JQ1 and DNA-protein cross-linked products were collected for ChIP assay. After BMS-986158 treatment (100 nM), the relative enrichment ratio of CDK9 and RNAP II increased by 7–8 times, and JQ1 (1 μM) showed a similar effect (Figure 3D,E). These results suggest that BMS-986158 can promote the recruitment of P-TEFb and activate RNAP II to the HIV-1 LTR.

### 2.5. BMS-986158 Inhibits Cell Cycle Progression

**Figure 4:**
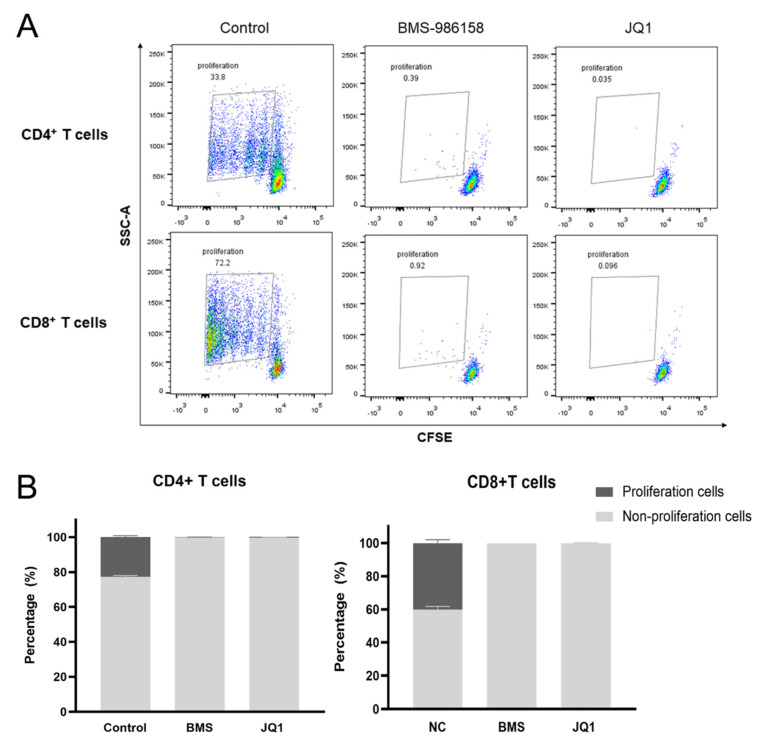
Effects of BMS-986158 on cell cycle. (**A**,**B**) BMS-986158 significantly inhibited proliferation of CD4^+^ and CD8^+^ T cells. PBMCs stained with carboxyfluorescein succinimidyl ester (CFSE) were treated with BMS-986158 (100 nM) or JQ1 (1 μM) for 5 d. Cells were then collected to determine CD4^+^ and CD8^+^ T cell proliferation by flow cytometry. All data represent the mean ± SD of three independent experiments. (BMS, BMS-986158).

BRD4 is an important regulator of gene expression and remains a prominent transcriptional vulnerability in human cancer [29]. In addition, some BET inhibitors are reported to influence the cell cycle [22]. Thus, we used PBMCs stained with carboxyfluorescein succinimidyl ester (CFSE) to detect the effects of BMS-986158 on cell proliferation. As shown in Figure 4A,B, the proportion of proliferating cells in untreated CD4^+^ T and CD8^+^ T cells accounted for 34.65 and 69.95%, respectively. However, the proportion of proliferating cells decreased to nearly 1% after treatment with BMS-986158, indicating that BMS-986158 possesses a strong capacity to inhibit the cell cycle of PBMCs.

### 2.6. BMS-986158 Synergizes with SAHA and Prostratin to Reactivate Latent HIV-1

**Figure 5:**
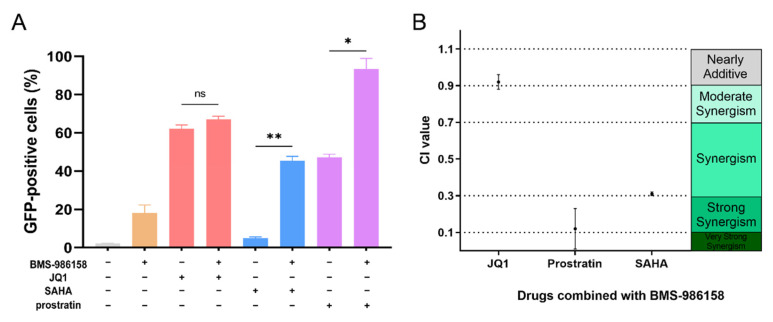
CI values of BMS-986158. (**A**,**B**) J-Lat cells were treated with gradient-diluted BMS-986158, prostratin, SAHA, and JQ1 alone or combined for 48 h. Cells were then harvested and percentage of GFP-positive cells was determined by flow cytometry. Effects of JQ1 (1 μM), SAHA (600 nM), and prostratin (10 μM) alone or combined with BMS (10 nM) on reactivation are shown in (**A**). CI value was analyzed using CalcuSyn software and summarized in (**B**). All data represent the mean ± SD of three independent experiments. Data were analyzed using one-way ANOVA followed by Dunnett’s T3 multiple comparisons test. The statistical significance between JQ1 and JQ1 + BMS-986158, SAHA and SAHA + BMS-986158, and prostratin and prostratin + BMS-986158 treatment group was showed in (**A**). Asterisks indicate statistical significance (* *p* < 0.05; ** *p* < 0.01; ns, no significance).

Although various LRAs have been discovered, their effects alone remain poorly studied [8]. To achieve HIV reactivation, the combined use of two or more LRAs may be an effective strategy. Here, the median-effect principle [30] was used to analyze the combined effects of BMS-986158 and SAHA or prostratin acting on different targets. As shown in Figure 5A, SAHA or prostratin combined with BMS-986158 showed a significant increase in reactivation activity compared to that when used alone. The combination index (CI) values of BMS-986158 + SAHA and BMS-986158 + prostratin were 0.31 and 0.12, respectively, thus indicating strong synergistic effects between each of these compound pairs (Figure 5B). The CI value of BMS-986158 + JQ1 was 0.92, thus representing a nearly additive interaction and indicating that BMS-986158 and JQ1 may act on the same target. Therefore, these results suggest that BMS-986158 shows synergism with other LRAs that act on different targets.

### 2.7. Effects of BMS-986158 on Latent HIV-1 Reactivation Are Not Impacted by Anti-HIV Drugs

**Figure 6:**
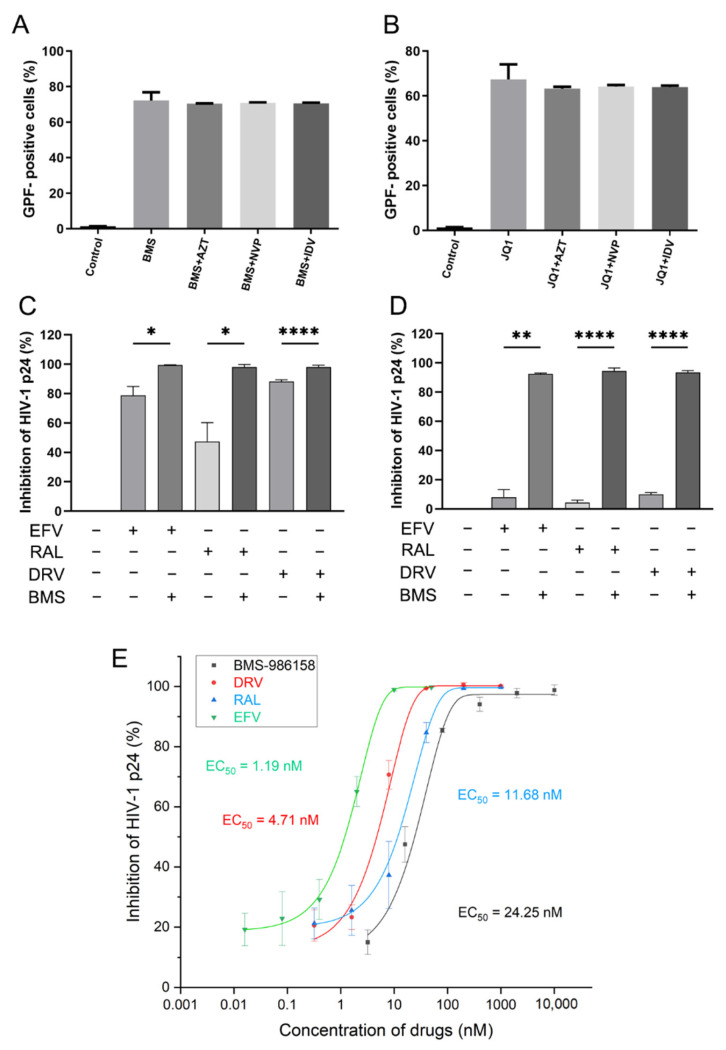
Interactions between BMS-986158 and anti-HIV-1 drugs. (**A**,**B**) Effects of anti-HIV-1 drugs on reactivation activity of BMS-986158 (100 nM) and JQ1 (1 μM). J-Lat cells were treated with BMS-986158 (**A**) alone or combined with zidovudine (AZT) (20 nM), nevirapine (NVP) (200 nM), or indinavir (IDV) (50 nM), or treated with JQ1 (**B**) alone or combined with AZT (20 nM), NVP (200 nM), or IDV (50 nM) for 48 h, with the percentage of GFP-positive cells then measured by flow cytometry. (**C**,**D**) BMS-986158 enhanced antiviral activity of anti-HIV-1 drugs. C8166 cells were infected with HIV-1_IIIB_ for 3 h and washed three times to remove free virus. Cells were resuspended in fresh RPMI-1640 medium and added to a 48-well plate with anti-HIV-1 drugs alone or combined with BMS-986158 (BMS-986158 (50 nM), efavirenz (EFV) (1 nM), raltegravir (RAL) (50 nM), and darunavir (DRV) (25 nM) in C; BMS-986158 (10 nM), EFV (0.5 nM), RAL (5 nM), and DRV (2.5 nM) in (**D**)). After 72 h, cell culture supernatant was collected to measure quantity of HIV-1 p24 by ELISA. (**E**) Antiviral activity of BMS-986158. C8166 cells were infected and washed as before and added to a 96-well plate with gradient-diluted BMS-986158, with HIV-1 p24 in the cell culture supernatant then measured by ELISA. All data represent the mean ± SD of three independent experiments. Data were analyzed using one-way ANOVA followed by Dunnett’s T3 multiple comparisons test. The statistical significance between EFV and EFV + BMS-986158, RAL and RAL + BMS-986158, and DRV and DRV + BMS-986158 treatment group was showed in (**C**,**D**). Asterisks indicate statistical significance (* *p* < 0.05; ** *p* < 0.01; **** *p* < 0.0001; BMS, BMS-986158).

To eliminate HIV via the “shock and kill” strategy, the efficacy of anti-HIV drugs cannot be disrupted by the use of LRAs. Here, three anti-HIV drugs (i.e., zidovudine (AZT), nevirapine (NVP), and indinavir (IDV), which are the most used ART drugs in China) with different targets were used to investigate their influence on the reactivation activity of BMS-986158. Results showed that BMS-986158 caused high levels of latent activation, regardless of whether it was combined with antiviral drugs or not (Figure 6A,B). These findings suggest that the reactivation activity of BMS-986158 is not affected by AZT, NVP, or IDV.

### 2.8. BMS-986158 Enhances Antiviral Activity of Anti-HIV Drugs

To assess the effects of BMS-986158 on the antiviral activity of anti-HIV drugs, we performed an antiviral assay to study changes in the anti-HIV activity of efavirenz (EFV), raltegravir (RAL), and darunavir (DRV) in combination with BMS-986158. As shown in Figure 6C, the inhibitory effects of EFV, RAL, and DRV against HIV-1 were ~50–80%, but increased to >95% when combined with BMS-986158 (100 nM). These results imply that BMS-986158 may enhance the antiviral activity of these three anti-HIV drugs. To verify these findings, we reduced the concentrations of BMS-986158 and the three anti-HIV drugs. Results showed that the inhibitory effects of EFV, RAL, and DRV against HIV-1 decreased to ~10% but increased to ~90% when combined with BMS-986158 (10 nM). Thus, BMS-986158 strongly enhanced the anti-HIV activity of the three drugs. We confirmed whether BMS-986158 alone can inhibit HIV-1 replication. Interestingly, BMS-986158 showed anti-HIV activity, with an EC_50_ value of 24.25 nM, while EFV, RAL, and DRV showed EC_50_ values of 1.19 nM, 11.68 nM, and 4.71 nM, respectively (Figure 6E).

To explore the mechanism underlying the effects of BMS-986158 on HIV-1, the activity of three key enzymes involved in HIV-1 replication was tested. However, results indicated that BMS-986158 did not inhibit the production of reverse transcription products and the activity of protease (Figure S1).

## 3. Discussion

HIV-1 infection cannot be cured due to the persistence of latent HIV-1 reservoirs [31]. The “shock and kill” strategy aims to eradicate these reservoirs via initial reactivation of latent HIV-1 (“shock”) and subsequent elimination using cART (“kill”) [6,32]. Various LRAs have been explored, with several PKC activators and HDAC inhibitors (e.g., SAHA, panobinostat, romidepsin, bryostatin, and disulfiram) undergoing clinical trials for the elimination or reduction of latent reservoirs, but with mostly unsatisfactory curative effects [33,34,35,36]. Here, BMS-986158, a new BET inhibitor for advanced solid tumors and hematological malignancies, showed potent reactivation effects on latent HIV-1 in the J-Lat cell model (EC_50_ of 32.83 nM). Both BMS-986158 and JQ1 directly inhibited BRD2 and BRD4 proteins in vitro, although the inhibitory effect of BMS-986158 was stronger than that of JQ1. The IC_50_ of JQ1 against BRD2/4 was 22.61–25.79-fold higher than that of BMS-986158, consistent with the EC_50_ of JQ1 in J-Lat cells, which was 18.28-fold higher than that of BMS-986158. Compared with other previously reported BET inhibitors, such as OTX015, UMB-136, RVX-208, PFI-1, and apabetalone [21,22,37], BMS-986158 showed the highest reactivation activity. Of note, the EC_50_ values of BMS-986158 and OTX015 were 17.28 and 4.34 times lower, respectively, than that of JQ1, while the reactivation activities of UMB-136, RVX-208, PFI-1, and apabetalone were all lower than that of JQ1. To further verify the reactivation effects of BMS-986158, we detected changes in CA HIV-1 RNA in PBMCs isolated from HIV-1-infected individuals. The expression of CA HIV-1 RNA in five of the 20 samples was under the detection limitation in the untreated group but reached detectable levels after treatment with BMS-986158 or JQ1. In another 15 samples, seven were reactivated by BMS-986158 and six were reactivated by JQ1. Thus, BMS-986158 and JQ1 appear to reactive samples with lower background expression (CA HIV-1 RNA expression level in untreated group), implying that BMS-986158 can reverse the deeply latent state of HIV. In the absence of Tat, RNAP II pauses on the HIV-1 LTR after initiation of HIV-1 transcription due to the transactivation response RNA element (TAR) [38]. Tat is the transactivator of HIV transcription and binds to TAR to promote transcriptional elongation [39]. P-TEFb can be recruited to Tat when it is present, and together they can bind to TAR [40]. This interaction enables CDK9 to phosphorylate the CTD of RNAP II as well as the DRB sensitivity-inducing factor and negative elongation factor, thus allowing RNAP II to initiate transcriptional elongation [41]. As the BRD4 and BRD2 proteins play suppressive roles in the transcriptional elongation of HIV-1, it is thought that their inhibition may result in the reactivation of latent HIV-1. Other BET inhibitors, such as JQ1 and OTX015, can reactivate latent HIV-1 by inducing phosphorylation of CDK9 [21,42]. To verify whether BMS-986158 exerts reactivation through the same mechanism, we examined changes in the activation and recruitment of P-TEFb and RNAP II after BMS-986158 treatment. Results demonstrated that BMS-986158 treatment significantly increased the phosphorylation of CDK9 in both the 42 kDa and 55 kDa CDK9 isoforms in a dose-dependent manner. Moreover, the recruitment of CDK9 and active RNAP II to the HIV-1 LTR also increased (about eight times) after treatment with BMS-986158. Notably, JQ1 showed a similar effect but at concentrations 10 times higher than that of BMS-986158.

Clinically suitable drugs should show high efficacy and low toxicity. Our results showed that BMS-986158 exerted little cytotoxicity in PBMCs, with a CC_50_ > 100 μM. In addition to cytotoxicity, several LRAs have been reported to initiate extensive T cell activation, which may aggravate T cell exhaustion and delay immune reconstitution in AIDS patients [43,44]. Thus, we tested the activation of CD4^+^ and CD8^+^ T cells after BMS-986158 treatment and found that BMS-986158 did not activate these cells. We also tested the effects of BMS-986158 on the expression of HIV-1 co-receptors and found that BMS-986158 did not significantly affect either the CXCR4 or CCR5 receptors, indicating that BMS-986158 does not increase the risk of infection by reactivated HIV-1.

Considering the unsatisfactory effects of LRAs in clinical trials to date, a single LRA may be insufficient to reactivate latent HIV-1. Combining two or more LRAs may be a feasible strategy as drug combinations with synergistic effects may enhance curative activity, reduce dosage requirements, and decrease potential drug resistance and side effects [45]. To explore potential combinations, we used the median-effect model to evaluate drug interactions between BMS-986158 and other LRAs. Results showed strong synergism between BMS-986158 and prostratin (PKC activator) and between BMS-986158 and SAHA (HDAC inhibitor) but showed nearly additive effects with JQ1. Drugs with the same target often show additive or antagonistic interactions [30], and thus the strong synergism between BMS-986158 and other LRAs with different targets suggests that BMS-986158 is suitable for combination therapy against latent HIV-1.

It is worth noting that in the “shock and kill” strategy, LRAs “shock” the system to reactivate the latent virus, while cART inhibits new infections, allowing the immune system to “kill” the reactivated cells [6]. Thus, interactions between LRAs and anti-HIV-1 drugs should not impact the safety of treatment. Here, we evaluated the interactions between BMS-986158 and three anti-HIV-1 drugs. Results showed that reactivation was not affected by the three anti-HIV-1 drugs with different targets. Surprisingly, we found that BMS-986158 enhanced the antiviral activity of the three anti-HIV-1 drugs in an acute HIV-1 infection model. We further confirmed that BMS-986158 alone exhibited antiviral activity, with an EC_50_ value of 24.25 nM (similar to its EC_50_ value for reactivation). To the best of our knowledge, this is the first report on the anti-HIV-1 activity of BET inhibitors in an acute infection model. To further explore the mechanism underlying the effects of BMS-986158 against HIV-1, we detected changes in the activity of three key enzymes involved in HIV-1 replication, i.e., reverse transcriptase, integrase, and protease, after BMS-986158 treatment. However, BMS-986158 did not show any inhibitory effects on these key enzymes. Although BMS-986158 down-regulated the expression of the CXCR4 and CCR5 receptors, the degree of CXCR4 and CCR5 down-regulation was low. The expression of CXCR4 and CCR5 decreased by about 5% after treatment with BMS-986158, which cannot explain the strong anti-HIV activity of BMS-986158. Thus, we hypothesize that BMS-986158 may act against HIV-1 replication via cell cycle arrest.

To date, only the HDAC inhibitor romidepsin has been reported to successfully reduce the viral reservoir in AIDS patients in clinical trials. Although no BET inhibitors have entered HIV latency associated clinical trials, BMS-986158 shows excellent synergistic effect with other LRAs and anti-HIV drugs and does not cause global immune activation. Thus, the performance of BMS-986158 in future clinical trials is eagerly awaited. Drawing on the successful experience of romidepsin, highly effective LRAs such as BMS-986158 combined with immunotherapy provide hope for the reduction or even clearance of latent HIV reservoirs.

This study had several limitations. The reason why some patient samples did not respond to BMS-986158 or JQ1 in the ex vivo experiments is unknown and needs to be addressed in future clinical applications. More importantly, our studies were performed in vitro, and thus further investigations are needed to verify the effectiveness of BMS-986158 in vivo.

## 4. Materials and Methods

### 4.1. Ethics Statement

Ethics approval for this study and consent processes were provided by the Ethics Committee of the Kunming Institute of Zoology, Chinese Academy of Sciences (Approval Number: SWYX-2006011, 2013016).

### 4.2. Compounds

BMS-986158, JQ1, and SAHA were purchased from MedChem Express (Monmouth Junction, NJ, USA). Prostratin was purchased from Sigma-Aldrich (Burlington, MA, USA). Zidovudine (AZT), efavirenz (EFV), nevirapine (NVP), raltegravir (RAL), indinavir (IDV), and darunavir (DRV) were purchased from Meilunbio^®^ (Dalian, China).

### 4.3. Cell Lines and Culture

The CD4^+^ T-lymphoid J-Lat A10.6 cell line (containing a full-length integrated HIV-1 genome with a GFP gene in place of the env gene) was kindly provided by Jian-Hua Wang (Guangzhou Institutes of Biomedicine and Health, Chinese Academy of Sciences). The ACH2, OM10.1, and C8166 cells were kindly provided by the National Institutes of Health (NIH, Manassas, VA, USA). The J-Lat A10.6 and C8166 cells were cultured in RPMI-1640 medium with 10% fetal bovine serum (FBS) (Invitrogen) at 37 °C in 5% CO_2_ in a humidified incubator. The ACH2 and OM10.1 cells were cultured in RPMI-1640 with 10% FBS and 50 μM IDV, with IDV removed before treatment with compounds.

### 4.4. Measurement of HIV-1 Latency Reversal In Vitro

The J-Lat A10.6 cells were stimulated with different compounds for appropriate times (typically 48 h) and then harvested. The percentage of reactivated cells (GFP-positive cells) was analyzed by flow cytometry and the concentration for 50% of maximal effect (EC_50_) was calculated. The ACH2 cells were treated with different compounds for 48 h. The level of HIV-1 p24 in the culture supernatant was then measured by enzyme-linked immunosorbent assay (ELISA) and the change in HIV-1 p24 was calculated.

### 4.5. Acquisition of PBMCs

For the reactivation assay, PBMCs were isolated from fresh whole blood of viremia-suppressed HIV-1-infected patients using density-gradient centrifugation with the Ficoll standard operating procedure (TBDscience, Tianjing, China). The PBMCs for cell viability, activation, and receptor detection were isolated from fresh whole blood of healthy HIV-1-negative donors as above.

### 4.6. Measurement of HIV-1 Latency Reversal Ex Vivo

PBMCs were treated with BMS-986158 or JQ1 for 24 h then collected for RNA extraction using Trizol reagent (Takara, Shiga, Japan). One-step quantitative reverse transcription polymerase chain reaction (qRT-PCR) was performed using a THUNDERBIRD^TM^ Probe One-Step qRT-PCR Kit (TOYOBO, Osaka, Japan) on a QuantStudio 5 Real-Time PCR System (ABI, Waltham, MA, USA) with forward (5′-CATGTTTTCAGCATTATCAGAAGGA-3′) and reverse primers (5′-TGCTTGATGTCCCCCCACT-3′) and probe (5′-FAM-CCACCCCACAAGATTTAAACACCATGCTAA-TAMRA-3′).

### 4.7. Cell Viability Assays

The PBMCs (5 × 10^5^ cells/mL) were added to 96-well plates with gradient-diluted compounds and incubated at 37 °C under 5% CO_2_ for 48 h, after which 20 μL of MTT (Sigma-Aldrich) was added. After incubation for 4 h, 100 μL of culture supernatant was removed and 100 μL of 12% sodium dodecyl sulfate (SDS)-50% *N*,*N*-dimethyl formamide (Sigma-Aldrich) was added. The plate was incubated at 37 °C overnight. Optical density (OD) was measured using an ELx800 reader (BioTek, Santa Clara, CA, USA) at 570 and 630 nm, and the 50% cytotoxicity concentration (CC_50_) was calculated.

### 4.8. Cell Proliferation Assay

Fresh PBMCs were resuspended in prewarmed phosphate-buffered saline (PBS) at a final concentration of 1 × 10^6^ cells/mL, followed by staining with 5 μM carboxyfluorescein succinimidyl ester (CFSE) (MedChem Express) at 37 °C for 10 min. Five volumes of ice-cold culture medium were added and incubated for 5 min on ice to quench staining. The PBMCs were washed three times with ice-cold culture medium and added to a 48-well plate at a final concentration of 1 × 10^6^ cells/mL. After 5 days of incubation, the cells were harvested, and cell proliferation was determined using flow cytometry in the FITC channel.

### 4.9. BRD2(1,2) and BRD4(1,2) Homogeneous Time-Resolved Fluorescence (HTRF) Assay

Serially diluted compounds were transferred to a 384-well plate, followed by 2 × protein and peptide mix and 2 × detection mix. The plate was shaken for 30 s and then incubated at room temperature for 1 h. The HTRF signals were determined using a Envision multilabel reader (Ex at 340 nm, Em at 615 and 665 nm) at Shanghai ChemPartner Co., Ltd. (Shanghai, China). Inhibition was calculated using the equation: Inhibitory (%) = (Max − Signal)/(Max − Min) × 100.

### 4.10. Antiviral Activity Assay

The C8166 cells (2 × 10^6^ cells/mL) were infected with HIV-1_IIIB_ (MOI = 0.1) for 3 h, then washed three times to remove free HIV-1_IIIB_. The cells were then plated in a 48-well plate (1 × 10^5^ cells/well) and treated with different concentrations of BMS-986158, EFV, RAL, and IDV. The cell culture supernatant was harvested at 72 h post-treatment and 5% triton X-100 was added to lyse the virus. The level of HIV-1 p24 was measured by ELISA.

### 4.11. ELISA for HIV-1 p24

A self-developed method was used to detect HIV-1 p24 as described previously [46]. Briefly, 96-well plates were coated with anti-mouse IgG Fc antibody overnight at 4 °C. After washing three times with PBS with Tween 20 (PBST) and blocking with 5% non-fat milk, mouse anti-HIV-1 p24 monoclonal antibodies were added. After 1 h of incubation at 37 °C and washing three times (PBST), the previously harvested supernatant was added and incubated for 2 h at 37 °C. The plates were then washed three times (PBST) and rabbit anti-HIV-1 p24 polyclonal antibodies were added. After 1 h of incubation at 37 °C and washing three times with PBST, goat anti-rabbit-horseradish peroxidase (HRP)-conjugated secondary antibodies were added, followed by 1 h of incubation at 37 °C. After again washing three times (PBST), o-phenylenediamine (OPD) substrate was used for chromogenic reaction and H_2_SO_4_ was used to stop the reaction. OD was measured at 490/630 nm using an ELx800 reader (BioTek).

### 4.12. Western Blotting

Western blotting was carried out as described previously [47]. The J-Lat A10.6 cells were treated with BMS-986158 (20, 100, and 500 nM) or JQ1 (1 μM) for 24 h and lysed using strong lysis buffer on ice for 10 min. Protein extract (20 μg) was then loaded on a 10% polyacrylamide gel and electroblotted onto a polyvinylidene fluoride (PVDF) membrane and blocked for 2 h. The membrane was then incubated with beta-tubulin (Abcam, Boston, MA, USA), CDK9 (CST), phosphor-CDK9 (pT186) (CST), or cyclin T1 (CST) antibodies at 4 °C overnight. After washing with TBST, corresponding HRP-labeled secondary antibodies were added and incubated at room temperature for 1 h, then washed again using TBST. Bands were visualized using an ECL western blotting system (Tanon, Shanghai, China).

### 4.13. Chromatin Immunoprecipitation (ChIP) Assay

The ChIP assay was performed using a SimpleChIP^®^ Enzymatic Chromatin IP Kit (Magnetic Beads) according to the manufacturer’s protocols (CST). Briefly, J-Lat cells (5 × 10^6^ cells/ mL) were treated with dimethyl sulfoxide (DMSO), BMS-986158 (100 nM), or JQ1 (1 μM) for 6 h. Cross-linking was achieved in 1% formaldehyde in medium for 10 min and reactions were stopped by the addition of glycine. Cells were washed twice using precooled PBS, resuspended in lysis buffer, and sonicated to obtain DNA fragments (150–900 bp). The DNA fragments were incubated with IgG (CST), anti-CDK9 Abs (Abcam), or anti-RNA Pol II CTD Ser2P Abs (Abcam) at 4 °C overnight. Immune complexes were retrieved by ChIP-grade protein magnetic beads for 2 h. After washing, reverse cross-linking was achieved using a mixer at 65 °C for 30 min. DNA was then extracted and purified and qPCR was performed using Taq PCR Master Mix (Takara) on a QuantStudio 5 Real-Time PCR System (ABI) with forward (5′-AGACTGCTGACATCGAGCTTTCT-3′) and reverse primers (5′-GTGGGTTCCCTAGTTAGCCAGAG-3′). Results from fragments obtained after incubation with different antibodies were normalized against input DNA and presented as fold-change relative to the DMSO control.

### 4.14. Combination Reactivation Assay

The BMS-986158, JQ1, prostratin, and SAHA compounds were added to 48-well plates alone or combined. The J-Lat cells were then added to the wells and the plates were incubated at 37 °C under 5% CO_2_ for 48 h. The percentage of reactivated cells was analyzed by flow cytometry. Data were analyzed according to the median-effect principle [48] using CalcuSyn software v1.0. Reactivation activity to different drug concentrations was input in CalcuSyn and the 50, 75, 90 and 95% effect doses (ED_50_, ED_75_, ED_90_, and ED_95_, respectively) were determined to calculate the combination index (CI) as a measure of drug interaction (CI < 0.9 indicates synergistic, CI = 0.9–1.1 indicates additive, and CI > 1.1 indicates antagonistic interaction). The degree of synergy is proportional to the CI value, thus we defined Cis ranging from 0.1 to 0.3 as strong synergy, from 0.3 to 0.7 as synergy, from 0.75 to 0.85 as moderate synergy, and from 0.85 to 0.9 as slight synergy. The CI value for drug combinations was calculated using the following formula: CI value = (1 × ED_50_ + 2 × ED_75_ + 3 × ED_90_ + 4 × ED_95_)/10.

### 4.15. Statistical Analysis

Statistical parameters, including the exact value of *n* and statistical significance, are reported in the figures and their associated legends. One-way ANOVA or Kruskal–Wallis tests were selected for data analysis according to the data distribution and homogeneity of variance, using SPSS version 20 and GraphPad Prism 8.0 software. Asterisks indicate statistical significance (*, *p* < 0.05; **, *p* < 0.01; ***, *p* < 0.001; ****, *p* < 0.0001; ns, no significance, *p* > 0.05).

## 5. Conclusions

In summary, we found that BMS-986158 is highly effective at reactivating latent HIV-1 in cell models in vitro and in cART-treated suppressive patient-derived PBMCs ex vivo via activation of P-TEFb and recruitment of P-TEFb and RNAP II to the HIV-1 LTR. The reactivation activity of BMS-986158 is strongly enhanced by prostratin and SAHA without inducing global T cell activation and is not affected by the three anti-HIV-1 drugs that target HIV-1 transcriptase, integrase, and protease. Moreover, BMS-986158 shows clear anti-HIV-1 activity in an acute infection cell model, possibly via cell cycle arrest. These results indicate that BMS-986158 is a potential candidate compound for the “shock and kill” strategy in HIV-1 treatment.

## Figures and Tables

**Table 1:** The effect of BMS-986158 on the expression of cell associated (CA) HIV-1 RNA.

Donors	CA HIV-1 RNA (Copies/μg)				
	Mock	BMS-986158		JQ1	
1	ND	51.31	▲	626.68	▲
2	ND	324.1	▲	34,767.24	▲
3	ND	408.54	▲	43.64	▲
4	ND	1698.66	▲	157.18	▲
5	ND	191,317.33	▲	30.95	▲
6	18.24	256.25	▲	47.04	▲
7	29.03	54,035.73	▲	ND	▼
8	50.96	4378.61	▲	6947.05	▲
9	151.11	1005.2	▲	1676.74	▲
10	154.8	ND	▼	81.24	▼
11	268.85	5906.85	▲	1148.21	▲
12	672.81	972.46	▲	4582.25	▲
13	1275.58	211.36	▼	688.28	▼
14	1357.75	272.8	▼	302.68	▼
15	1528.55	2317.63	▲	813.12	▼
16	2384.24	287.39	▼	939.99	▼
17	4359.75	3.42	▼	5408.31	▲
18	6711.11	9.23	▼	2372.51	▼
19	9190.65	8532.24	▼	2857.87	▼
20	63,438.38	4198.45	▼	13,307.23	▼

(ND, Not determined; ▲, Up-regulated; ▼, Down-regulated).

## Data Availability

Data is contained within the article and Supplementary Materials.

## Supplementary Materials

The following are available online at https://www.mdpi.com/article/10.3390/ph15030338/s1, Figure S1: The effect of BMS-986158 on HIV-1 replication.
