# Supplementary material for: Bromodomain and Extra-Terminal Inhibitor BMS-986158 Reverses Latent HIV-1 Infection In Vitro and Ex Vivo by Increasing CDK9 Phosphorylation and Recruitment
Source: Pharmaceuticals (Basel). 2022 Mar 10;15(3):338. doi: 10.3390/ph15030338 (PMC8952190; doi:10.3390/ph15030338)
Supplement: Supplementary file 1 [file pharmaceuticals-15-00338-s001.zip › pharmaceuticals-1564230-supplementary.pdf]

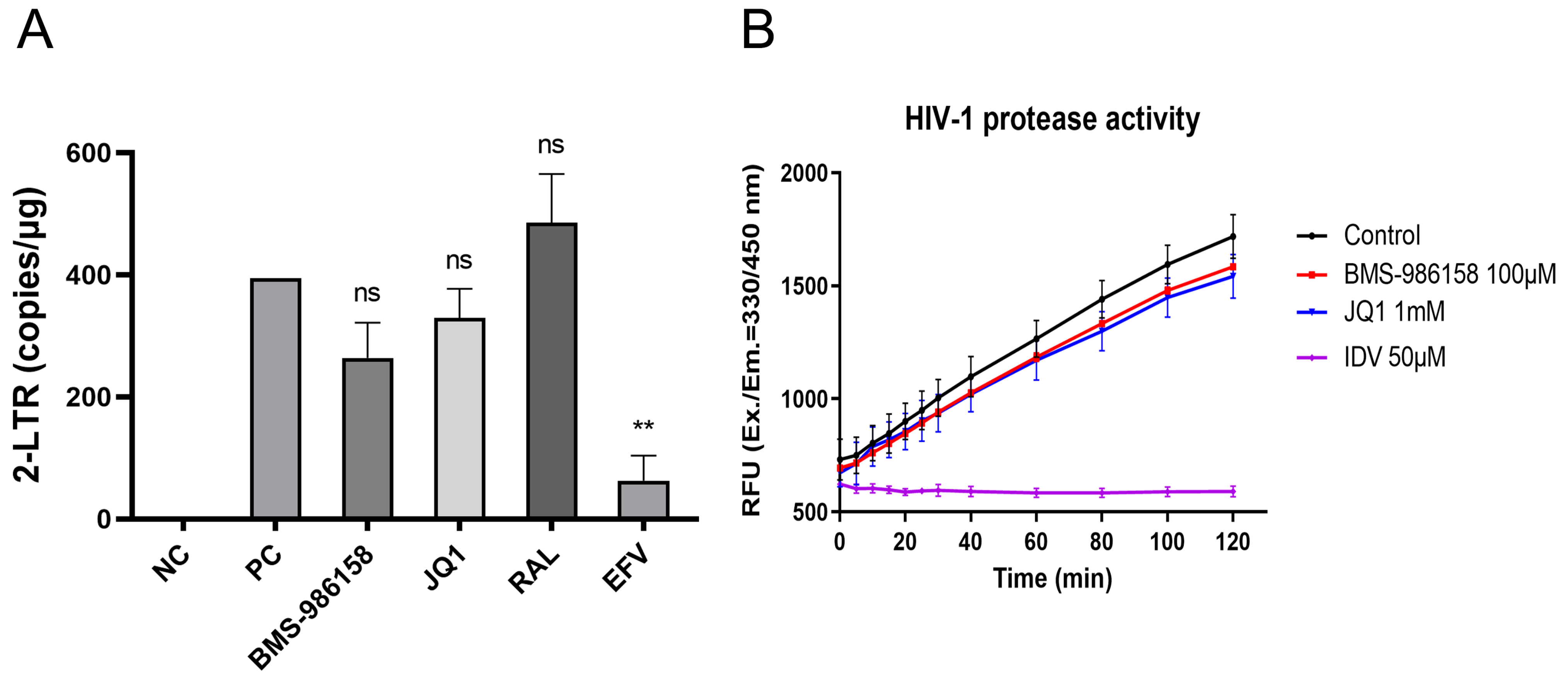

Figure S1. the effect of BMS-986158 on HIV-1 replication. (A) BMS-986158 did not inhibit the transcription of HIV-1. HIV-1_{IIIB} acute infected C8166 cells were treated with BMS-986158 (100 nM), JQ1(1 µM), raltegravir (RAL) (500 nM) or efavirenz (EFV) (100 nM) for 24 h and cell were harvested for total DNA extraction. The expression of transcription production was determined by qPCR. **(B)** BMS-986158 did not inhibit the HIV-1 protease activity. Different concentration of compounds were added into 96-well plate containing protease and substrate and the fluorescence value was monitored with a microplate reader.
